# A Hybrid Method to Improve the BLE-Based Indoor Positioning in a Dense Bluetooth Environment

**DOI:** 10.3390/s19020424

**Published:** 2019-01-21

**Authors:** Ke Huang, Ke He, Xuecheng Du

**Affiliations:** Innovation and Development Department, Sichuan Energy Internet Research Institute, Tsinghua University, Chengdu 610000, China; huangke10@foxmail.com (K.H.); heke@tsinghua-eiri.org (K.H.)

**Keywords:** indoor positioning, Bluetooth Low Energy, dense Bluetooth environment, trilateration, dead reckoning, Kalman filter

## Abstract

Indoor positioning using Bluetooth Low Energy (BLE) beacons has attracted considerable attention after the release of the BLE protocol. A number of efforts have been exerted to improve the performance of BLE-based indoor positioning. However, few studies pay attention to the BLE-based indoor positioning in a dense Bluetooth environment, where the propagation of BLE signals become more complex and more fluctuant. In this paper, we draw attention to the problems resulting from the dense Bluetooth environment, and it turns out that the dense Bluetooth environment would result in a high received signal strength indication (RSSI) variation and a longtime interval collection of BLE. Hence, to mitigate the effects of the dense Bluetooth environment, we propose a hybrid method fusing sliding-window filtering, trilateration, dead reckoning and the Kalman filtering method to improve the performance of the BLE indoor positioning. The Kalman filter is exploited to merge the trilateration and dead reckoning. Extensive experiments in a real implementation are conducted to examine the performance of three approaches: trilateration, dead reckoning and the fusion method. The implementation results proved that the fusion method was the most effective method to improve the positioning accuracy and timeliness in a dense Bluetooth environment. The positioning root-mean-square error (RMSE) calculation results have showed that the hybrid method can achieve a real-time positioning and reduce error of indoor positioning.

## 1. Introduction

Until now, the GPS-based positioning system has successfully solved the outdoor localization and navigation problems. However, people spend more than 80% to 90% of their time indoors. It is estimated that the indoor positioning market will grow to $4.4 billion by 2019 with strong demand in healthcare, retail, hospitality, travel and other sectors [1]. GPS, which has revolutionized outdoors localization, has proven ineffective for indoor environments due to the lack of signal coverage. Hence, several alternative approaches for indoor positioning systems were applied: Wi-Fi based positioning systems [2], Bluetooth Low Energy (BLE) based positioning systems [3], Radio Frequency Identification (RFID)-based positioning systems [4], Ultra-Wide Band (UWB)-based positioning systems [5], Visible Light Communication (VLC)-based positioning systems [6], sensors-aided positioning systems [7,8]. These methods are based on different information sources like wireless communication technologies or sensor measurements. However, each of them has disadvantages, such as low precision, unreliability, high complexity or high hardware cost. For example, the Wi-Fi-based solutions have limitations due to the limited numbers of access points (APs) and their inflexibility in deployment, resulting in poor accuracy, from 5 to 15 m. In the case of RFID-based solutions, it has the best accuracy among all the technologies (error below 0.1 m) and needs no battery within its lifetime, but the short range (below 1 m) and the extensive and expensive installation of large amounts of readers restricts its use. Although UWB-based solutions have good accuracy (error below 0.3 m) and an up to 150 m range, the high-power consumption and high cost are its main disadvantages.

Besides, none of these technologies are a completed solution to fit all indoor positioning needs and there is no single technology that can provide reliable indoor positioning. Therefore, many researchers have tried to fuse two or more kinds of above technologies to design a robust and low-cost indoor localization method. In Ref. [9] Chen et al. carried out a Wi-Fi PDR (Pedestrian Dead Reckoning) integrated method to a real-time positioning for targets in 3D scenes. A combined Wi-Fi and BLE positioning system were presented to improve the accuracy and precision of indoor positioning with low deployment density [10]. A hybrid indoor positioning method for asset positioning by using BLE and Wi-Fi achieved 90% accuracy within 1.21 m [11]. BLE is also integrated with PDR to achieve a positioning accuracy of less than 1 m [12].

Most of the research and applications refer to systems using either BLE or Wi-Fi technologies, since they performed better in terms of quality and cost. BLE is a recently developed wireless technology, which improves Bluetooth technology, consumes less energy and has a long lifetime. As it is inexpensive, low-energy and easy to install, it has been widely employed for various purposes, like localization, proximity detection and interaction, active sensing, etc. [13]. The indoor positioning is one of the most important applications of BLE and positioning by BLE can achieve sub-meter accuracy [14]. Received Signal Strength Indicator (RSSI) is the most common way for BLE to estimate the position of the device being tracked. Generally, the most common methods of positioning are the trilateration algorithm and fingerprinting algorithm. Trilateration algorithm is the method of determining absolute or relative locations of points by measuring the distances using the geometry of circles, spheres or triangles [15]. In Ref. [12], Robesaat et al. proposed a positioning method based on fusing trilateration and dead reckoning with Kalman filtering as a position fusion algorithm, to achieve a positioning accuracy of less than 1 m. The fingerprinting algorithm relies on a previously-constructed radio map of BLE signals, in which measurements are collected from the environment and calibrated into specific features, called fingerprints, which describe a part of the environment [16]. In Ref. [17], Danis et al. developed a method to render indoor localization and tracking practical with mean of BLE RSSI fingerprinting. Generally, the fingerprinting-based positioning with dense fingerprint locations outperforms the trilateration-based positioning in terms of reliability and precision. However, compared to trilateration-based positioning, the fingerprinting-based positioning needs dense BLE deployment and high time-cost preparation work. Besides, the fingerprinting-based positioning is very susceptible to any environmental change which may cause the radio map to change [18].

Moreover, there are a lot of Bluetooth devices for achieving intellectualized control in modern buildings such as Bluetooth lamps, Bluetooth audio, and Bluetooth transmitters. These Bluetooth devices broadcast their Bluetooth signals incessantly resulting in a dense Bluetooth environment. The environmental conditions are very variable and therefore may affect accuracy and precision in relation to the actual location [3]. Most of the existing research has focused on the solutions that combined different supplementary means (Wi-Fi, PDR, etc.) to improve the accuracy of the BLE-based indoor positioning, since the RSSI of BLE is unreliable in distance estimation. However, few studies pay attention to the BLE-based indoor positioning in a dense Bluetooth environment, where the propagation of BLE signals become more complex and more fluctuant.

The distance estimation error and the signal timeliness become more severe when multiple Bluetooth sources are present within a small area, since a smart phone or a RSSI receiver might be unable to process all the signals within a short scanning period. It was proved that each of the 10 static beacons requires less than 1 s to be detected under a sparse environment, while the detection time can take more than 5 s under a dense environment [13]. A long interval collection or a partial signal collection may result in a poor accuracy of indoor positioning. In addition, with the development of Bluetooth smart home devices and the spread of Bluetooth built-in consumer electronics, more and more Bluetooth signals are present in the indoor environment, forming a dense Bluetooth environment. It is important and urgent to develop a BLE-based indoor positioning solution suitable for dense Bluetooth environments.

In this work, a hybrid approach of BLE-based indoor positioning using a set of pre-deployed BLE beacons and smart mobile devices was proposed for achieving high accuracy in a real implementation of a dense Bluetooth environment. Numerous exiting indoor localization methods, namely trilateration and PDR, were applied. Moreover, Kalman filtering was adopted as a position fusion algorithm to improve accuracy, which integrates the advantage of trilateration and PDR and relives their weaknesses as well. Finally, the method to a real implementation of dense Bluetooth environment was applied and the practicability was verified.

The remainder of this paper is divided into six parts as follows. Section 2 defines the problem resulting from the dense Bluetooth environment and the overview of our BLE-based indoor positioning system. Section 3 elucidates the BLE propagation model and the trilateration algorithm. Section 4 investigates the PDR method, followed by the proposed hybrid approach with Kalman filtering to improve the accuracy in a dense Bluetooth environment in Section 5. Section 6 illustrates the use of the hybrid approach in a real implementation of high dense environment and verifies the practicability of the method. Finally, Section 7 draws the conclusions.

## 2. Overview

In this section, the problem arising from dense Bluetooth environments is defined and the overview of the solution to the problem is proposed.

### 2.1. Problem Description

**Figure 1:**
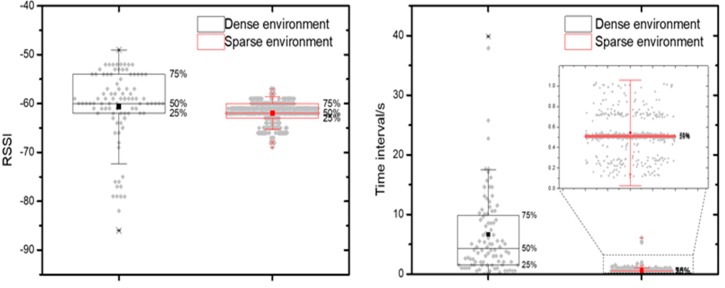
Received Signal Strength Indicator (RSSI) value and the scanning time interval of the Bluetooth Low Energy (BLE).

Despite the fact that the Bluetooth Classic and the BLE have a different number of channels (Bluetooth Classic has 79 channels and BLE has 40 channels) [19], a smartphone will listen to all Bluetooth Classic and BLE signals during the scanning period indiscriminatingly. This fact poses a troublesome situation for BLE positioning in a dense Bluetooth environment, which needs the specific BLE signals to calculate the position of the target. Additionally, a lot of the unwanted and distracted Bluetooth signals in the environment will affect the scanning of the wanted and specific BLE signals. In order to measure the effect of the dense Bluetooth environment, the RSSI value and the scanning time interval of the BLE were measured respectively in the dense environment (with 10 Bluetooth Classic sources and three BLE sources) and sparse environment (without any other Bluetooth sources) within a 10-min continuous scanning time and a constant distance of 1.0 m from the BLE module. The RSSI value and the scanning time interval are shown in the Figure 1 and Table 1.

The result shows that within 10 min the smartphone received the target BLE signal 93 times in a dense environment, but 1109 times in a sparse environment. The significant difference was also reflected in the scanning time interval, with an average 6.652 s time interval in a dense environment and an average 0.542 s time interval in a sparse environment. Besides, longer scanning time interval variation was observed in a dense environment than that in a sparse environment. Although the average RSSI values of both situations showed little difference, the RSSI standard deviation and the difference between the maximum value and the minimum value in both cases are significantly different.

All of the results revealed that a high Bluetooth density of the environment would lead to a high RSSI variation and a long collection time interval of BLE. The high RSSI variation will result in an unreliable distance estimation and a low accuracy of positioning. Additionally, a long-time interval collection of BLE will lead to poor timeliness of positioning.

### 2.2. Overview of Solution

**Figure 2:**
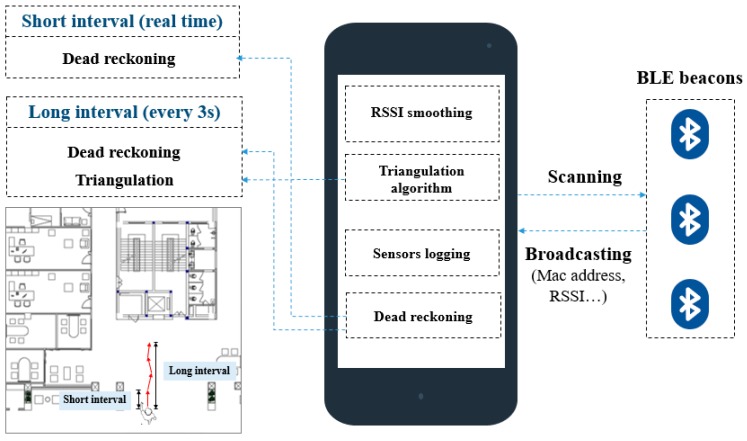
The overview of BLE positioning solution.

As mentioned above, the high RSSI variation and long scanning interval stand against obtaining an accurate indoor positioning. Therefore, a hybrid approach overcoming the problem in the dense Bluetooth environment was proposed, as shown in Figure 2. Several BLE beacons were fixed in specific positions in the ceiling with a distance of 4 to 6 m between every two beacons. Every BLE beacon was broadcasting their Mac address, RSSI, major, minor, etc. During the positioning, a mobile device was scanning the BLE advertisements to locate itself by triangulation algorithm and activating its embedded sensors to locate itself by dead reckoning. The RSSI values of BLE were collected to compute the location of the mobile device by triangulation algorithm. Due to the high RSSI variation and a long collection interval of BLE resulting from the dense Bluetooth environment, the indoor positioning system was divided into two positioning phases. During the short interval, the dead reckoning was used to determine the location of the mobile device. However, the triangulation algorithm was exploited to correct the deviation of the dead reckoning caused by accumulative sensing errors in the long interval. To achieve that, a Kalman filter was used as a fusion center to merge the two obtained positions from the two algorithms.

## 3. Trilateration Positioning Method

In this section, the BLE propagation model and the BLE-based triangulation method are investigated. Due to the issues of high RSSI variation in the dense Bluetooth environment, a RSSI smoothing method is described first. Then, a BLE propagation model is presented to estimate the distance from the BLE modules in the dense Bluetooth environment.

### 3.1. RSSI Smoothing Method

A more accurate distance estimation between mobile device and BLE beacon is the key to position calculation with triangulation algorithm, which relies on a low RSSI variation. Therefore, it is necessary to smoothen the RSSI signal before the application of triangulation algorithm. A moving average filter was used to smoothen the received RSSI signals as follows:

(1) $$smooth\_RSSI_{N}=0.1\times RSSI_{N}+0.9\times RSSI_{N-1}.$$

**Figure 3:**
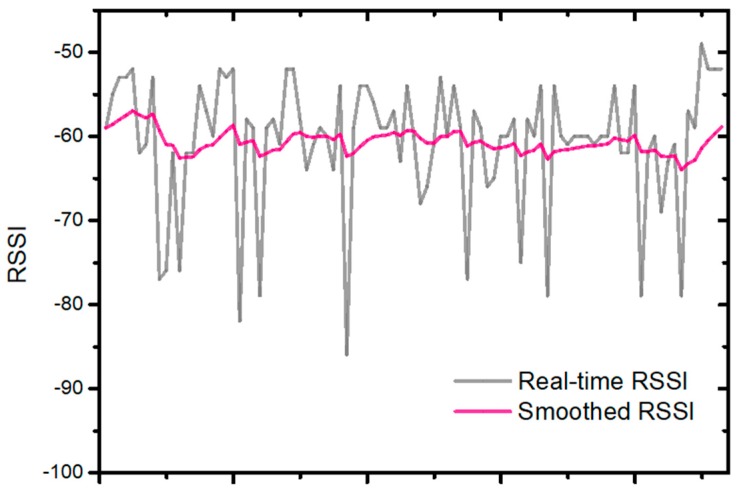
The curves of smoothed RSSI and real-time RSSI.

The real-time received RSSI signals and the smoothed RSSI signals are shown in Figure 3. The real-time RSSI signals varied a lot at a constant distance of 1.0 m away from the BLE beacon due to the dense Bluetooth environment. It turned out that the moving average filter reduced the RSSI fluctuation effectively. As a result, a moving average filter was applied for all following RSSI filtering in this work.

### 3.2. BLE Propagation Model

The RSSI value decreases with the distance increase away from the BLE beacon and the environment will affect the propagation of the BLE signal, where the RSSI is highly affected by multipath and fading phenomena. The most used model to model the relationship between measured RSSI and corresponding distances is the log-distance path loss model as follows [20]:

(2) $$RSSI(d)=RSSI(d_{0})-10n\times \log_{10}\left(\frac{d}{d_{0}}\right)+X_{\sigma },$$

where *RSSI*(*d*_0_) is the RSSI reference value measured at the distance *d*_0_. The parameter *n* is the path loss exponent, indicating the rate of increasing path loss related to the distance. The *d* represents the actual distance to the beacon, while *RSSI*(*d*) is the measured RSSI value at the distance of *d*. The *X_σ_* represents a Gaussian random variable, with zero mean, caused by shadow fading [21].

In this work, *d*_0_ was set to 1 m, and the *RSSI*(*d*_0_) was the measured RSSI value at a distance of 1 m to the BLE beacon. Since the experiment environment had no large obstacles, shadow fading was not expected, and *X_σ_* was set to zero. To get the BLE propagation model, the RSSI values of three BLE beacons were measured at different distances respectively under the same environment. At each distance, a 2-min RSSI collection with each BLE beacon was conducted. With the measured RSSI values and the pre-known distances, the path loss exponent *n* can be calculated by the following equation:

(3) $$n=\frac{RSSI(d_{0})-RSSI(d)}{10\times \log_{10}\left(10\right)}.$$

**Figure 4:**
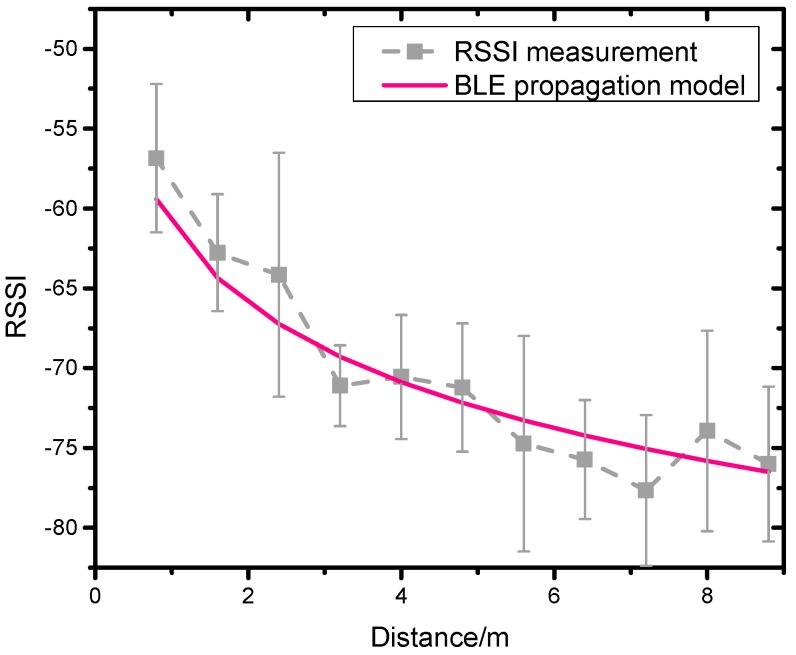
The RSSI measurement value and BLE propagation model value.

Table 2 shows the path loss exponent *n* of three BLE beacons in the dense Bluetooth environment and Figure 4 shows the measured RSSI values and the theoretical RSSI values from the BLE signal propagation model regarding the reference distance. The average value of *n* = 1.64 was used for further measurements.

### 3.3. Triangulation Method

Once we got the RSSI values of the pre-known BLE beacons, the distances to the beacons can be calculated by the BLE propagation model. Then, the position of the RSSI receiver was calculated with the method of triangulation algorithm. At least three of the reference BLE beacons with pre-known positions were required in this method. In this method, each reference beacon forms a circle around itself with the radius of the distance to the receiver theoretically and the position of the unknown receiver corresponds with the intersection of these three circles [12]. The distances in a plain between the *i*th known BLE beacon and the unknown receiver can be expressed by the following equation:

(4) $${d_{i}}^{2}={\left(x-x_{i}\right)}^{2}+{\left(y-y_{i}\right)}^{2},$$

where the coordinate (*x_i_*, *y_i_*) represents the position of the *i*th BLE beacon while the (*x*, *y*) is the coordinate of the receiver. Furthermore, in most cases, more than three reference beacons are available and the position of target receiver can be expressed in the form of

(5) $$A\vec{x}=b,$$

with:

(6) $$A=\left[\begin{array}{cc} 2(x_{1}-x_{n}) & 2(y_{1}-y_{n})\\ \vdots & \vdots \\ 2(x_{n-1}-x_{n}) & 2(y_{n-1}-y_{n}) \end{array}\right],$$

(7) $$\vec{x}=\left[\begin{array}{c} x\\ y \end{array}\right],$$

(8) $$b=\left[\begin{array}{c} x_{1}^{2}-x_{n}^{2}+y_{1}^{2}-y_{n}^{2}+d_{n}^{2}-d_{1}^{2}\\ \vdots \\ x_{n-1}^{2}-x_{n}^{2}+y_{n-1}^{2}-y_{n}^{2}+d_{n-1}^{2}-d_{1}^{2} \end{array}\right].$$

Additionally, the equation can be solved as the least-squares problems as following:

(9) $$\vec{x}={\left(A^{T}A\right)}^{-1}(A^{T}b).$$

## 4. PDR Method

This section mainly investigated the dead reckoning method based on the internal sensors of a mobile device and presented the PDR algorithm based on the mobile sensors, followed by a detailed explanation of the method of detecting steps. Finally, the heading direction was analyzed. A smartphone was utilized for the following experiments. 

### 4.1. PDR Algorithm

According to the PDR method, the accelerometer, the magnetic sensor and the gyroscope embedded in a smart phone will be combined into an inertial measurement unit to locate the position of the mobile terminal based on the following formula:

(10) $$\{\begin{array}{c} x_{i+1}=x_{i}+sl\times \cos\alpha_{i}\\ y_{i+1}=y_{i}+sl\times \sin\alpha_{i} \end{array},$$

where (*x*, *y*) indicates the coordinates of the mobile position, *sl* denotes the step length and α represents the heading angle, thus the PDR algorithm mainly consists of the gait detection, step length and heading direction. Furthermore, one person’s step length was assumed as 0.70 m in this work.

### 4.2. Step Detection

**Figure 5:**
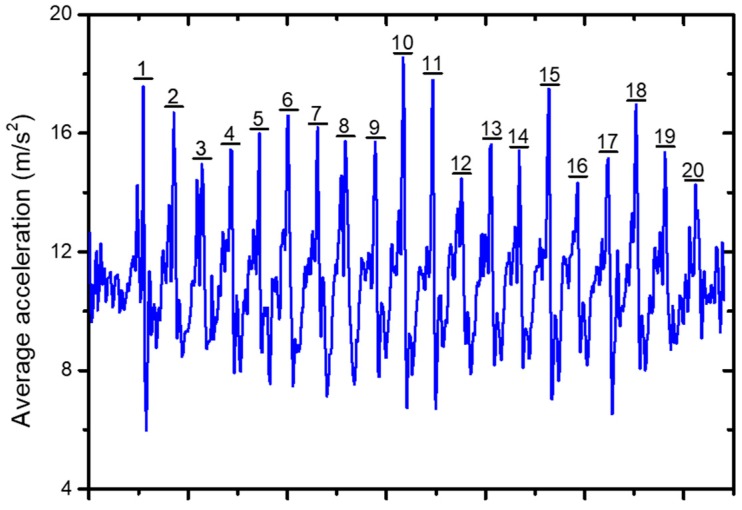
Average acceleration record of 20 steps.

The accelerometer embedded in the smart phone can detect the occurrence of a step [22,23]. Both of the vertical and horizontal signals of the accelerometer graph provide the number of steps taken. The measurement of the acceleration was performed, and Figure 5 shows the wave form of the average acceleration recorded during 20 steps, where the average acceleration represents the root mean square value of the acceleration force along the *x*-, *y*- and *z*-axis.

The detection algorithm can be applied to the accelerometer graph and a number of identification methods can be used. One of the simplest ways is to identify a step through a high peak. However, due to the multiple peaks and the amplitude variation of measurements, the high peak is not reliable enough. Alternatively, an improved method uses the fact that a high peak is always followed by a low peak during a taken step [24]. Therefore, a high and low threshold were set to recognize the peaks and a step was counted at a high peak followed by a low peak. Nevertheless, an unintended movement of the mobile can result in an incorrect step count. To solve the problem, a step identification method characterized by duration time was proposed [25]. Additionally, a typical time between the high and the low peak takes about 150–400 ms, which can be the additional constraint to the step characterization.

In our step detection method, the high peaks and the low peaks of the acceleration graph were identified, and a step was detected when two conditions were all met. Firstly, the difference value of high peak and adjacent low peak must exceed the set threshold. Secondly, the time between the high peak and the adjacent low peak must be between 150 and 400 ms. To find the best threshold, step measurements in different thresholds were carried out. The user walked 20 steps in each test, and the results are shown in Table 3. It turned out that the lower the threshold was, the more sensitive the step count method was. when the threshold was 3, the error rate was the lowest.

### 4.3. Heading Estimation

The orientation sensor was used to get the heading direction of the smart phone. To determine the accuracy of the heading, eight different heading direction tests were carried out. In each direction, 1000 single measurements were recorded. The results of the tests are shown in Table 4.

The results showed that the average error is less than 2°. The reason may be: the user took a step along the *x*-axis, and it was expected that the *x*-value is increased by 0.70 m, and the *y*-value did not change. If the heading measurement is measured incorrectly by 2°, the distance error will be:

(11) $$x_{i+1}-x_{i}=0.70\times \cos\left(2{}^{\circ}\right)=0.69999\;m,$$

(12) $$y_{i+1}-y_{i}=0.70\times \sin\left(2{}^{\circ}\right)=0.00389\;m,$$

(13) $$\varepsilon =\sqrt{{\left(0.70-0.70\times \sin\left(2{}^{\circ}\right)\right)}^{2}+{\left(0-0.70\times \cos\left(2{}^{\circ}\right)\right)}^{2}}=0.0039\;m.$$

It showed that a deviation of 2° leads to only a distance error of less than 1 cm, which is acceptable for the further calculation.

## 5. Hybrid Indoor Positioning

**Figure 6:**
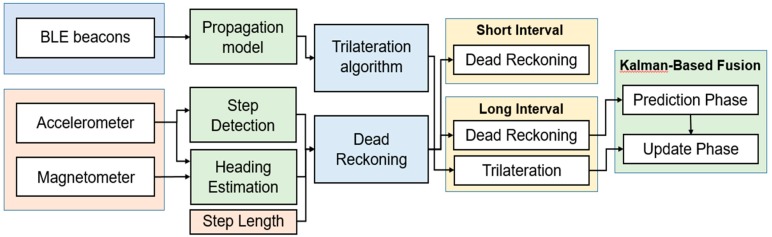
The processing pipeline of the hybrid indoor positioning.

**Figure 7:**
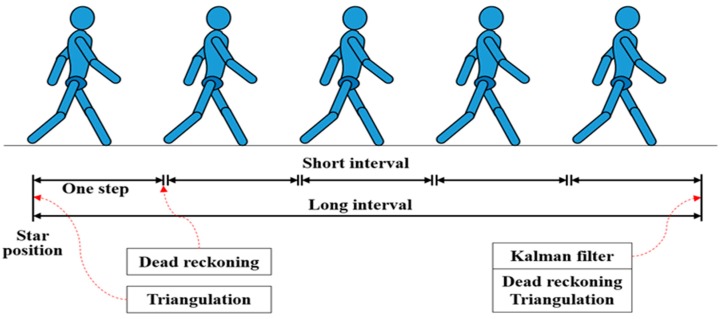
Diagram of two positioning phases.

In this section, the hybrid indoor positioning method combined with Trilateration algorithm and dead reckoning is illustrated. The hybrid positioning method centered on overcoming the high RSSI variation and a long collection time interval of BLE resulting from the dense Bluetooth environment and exploiting the advantages of Trilateration algorithm and dead reckoning, but avoiding possible disadvantages, as depicted in Figure 6. As mentioned before, the indoor positioning system was divided into two positioning phases, as shown in Figure 7. During the short interval, the dead reckoning was used to determine the location of the mobile device. However, during the long interval, the triangulation algorithm was exploited to correct the deviation of the dead reckoning caused by accumulative sensing errors. To achieve that, a Kalman filter was used as a fusion center to merge the two obtained positions from the two algorithms.

### 5.1. Two Positioning Phases

As mentioned above, a lot of the unwanted and distracted Bluetooth signals in the environment will affect the scanning of the wanted and specific BLE signals in a dense Bluetooth environment. Moreover, an average 6.652 s time interval was needed for one BLE signal collection in the dense environment, which means that the frequency of one BLE beacon signal collected by a phone was 0.15 Hz. In this indoor positioning system, there were eight BLE beacons distributed in the ceiling, which means that the frequency of the one of the eight beacons signals collected by a phone was 1.2 Hz. The trilateration algorithm took at least three BLE signals to calculate the position of the target and it would take at least 2.5 s to collect enough BLE signals feeding the trilateration algorithm to get the position. However, during the 2.5 s interval, one person possibly moves a long distance and the result from the trilateration algorithm loses timeliness leading to a wrong positioning estimation. Therefore, the dead reckoning will contribute to the real time positioning estimation. Dead reckoning produces highly accurate position fixes at the beginning of the measurements. Nevertheless, the dead reckoning performance continuously drops due to accumulative sensing errors. Consequently, it was necessary to divide the indoor positioning system into two positioning phases in a dense Bluetooth environment. During the short interval, the dead reckoning was used to determine the location of the mobile device. During the long interval, the triangulation algorithm was exploited to correct the deviation of the dead reckoning caused by accumulative sensing errors. To get the enough BLE signals for trilateration algorithm, the long interval was set to 3 s.

### 5.2. Kalman-Based Fusion

During the long interval phase, a Kalman filter was chosen to integrate the trilateration algorithm and dead reckoning [12]. In the Kalman filter, the state model is obtained from the dead reckoning method and the state equation can be expressed as:

(14) $${\hat{x}}_{k}=(F\times {\hat{x}}_{k-1})+B\times \sum_{i=1}^{N}u_{i},$$

where the state vector ${\hat{x}}_{k}={\left(x,y\right)}^{T}$ represents the coordinates of the target from the dead reckoning. *F* and *B* are identity matrices, and *N* is the total amount of counted steps during the long interval phase. While, $u_{i}=sl\times {\left(\cos\alpha_{i},\sin\alpha_{i}\right)}^{T}$ is the coordinate change derived from *i*th step. In this case, *sl* is the step length and $\alpha_{i}$ is the heading direction. Therefore, the coordinate change resulting from the dead reckoning method during the long interval phase can be expressed as:

(15) $$u_{k}=\sum_{i=1}^{N}sl\times \left[\begin{array}{c} \cos\alpha_{i}\\ \sin\alpha_{i} \end{array}\right].$$

The measurement model is obtained from the trilateration positioning method. The measure model can be expressed as:

(16) $${\hat{z}}_{k}=H{\hat{x}}_{k},$$

where ${\hat{z}}_{k}={\left(x_{k},y_{k}\right)}^{T}$ represent the coordinate of the target from the trilateration positioning, and the *H* is identity matrix.

Once the time interval reaches 3 s, the Kalman filter updates the position with a prediction and an update phase. In the prediction phase, a position can be predicted as following:

(17) $${\hat{x}}_{k}^{-}={\hat{x}}_{k-1}+\sum_{i=1}^{N}sl\times \left[\begin{array}{c} \cos\alpha_{i}\\ \sin\alpha_{i} \end{array}\right],$$

(18) $$P_{k}^{-}=FP_{k-1}F^{T}+Q,$$

where *Q* is the covariance of the noise from the dead reckoning method. In the update phase, the positions from the both methods can be merged as following:

(19) $$K_{k}=P_{k}^{-}\times H^{T}{\left(HP_{k}^{-}H^{T}+R\right)}^{-1},$$

(20) $${\hat{x}}_{k}={\hat{x}}_{k}^{-}+K_{k}({\hat{z}}_{k}-H{\hat{x}}_{k}^{-}),$$

(21) $$P_{k}=(I-K_{t}H)P_{k}^{-},$$

where the *K* is the Kalman gain and *I* is identity matrix. *R* is the covariance of the noise from the trilateration positioning method, and ${\hat{x}}_{k}$ represents the merged position. Different values for *R* and *Q* were experimentally tested, and the best results were obtained by using *R* = 4 and *Q* = 0.1.

## 6. Implementation of the Hybrid Method

In this section, the performance of the proposed hybrid method was evaluated in a dense Bluetooth environment. A mobile device was used to carry out the tests.

### 6.1. Test Scenario

**Figure 8:**
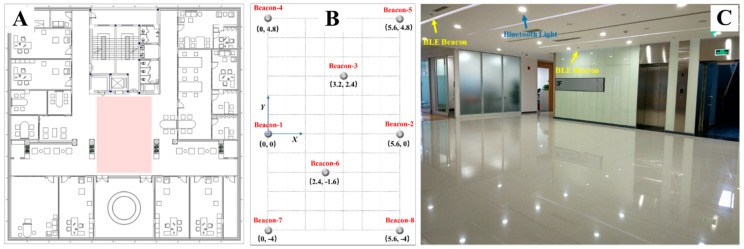
Layout of the test scenario.

To evaluate the performance of the proposed algorithm, we conducted experiments in the middle of the office floor (the red square area), depicted in Figure 8A. The area size was 5.6 × 8.8 m^2^. There were 18 Bluetooth lights in the square area they broadcasted their Bluetooth signals incessantly resulting in a dense Bluetooth environment, as shown in Figure 8C. Eight BLE beacons were placed in the square area, depicted in Figure 8B. Each of the beacons was placed in the ceiling, at a height of 2.7 m. The tester walked with a smartphone in the test area following the predefined path. The distance between two beacons was 4 to 6 m, such that the square was entirely covered by BLE signals. Since a 2D trilateration method was applied, the vertical distance Δh between the beacon and the mobile was required to get the horizontal distance $d_{h}$ between the beacon and the mobile. Hence, the horizontal distance $d_{h}$ in terms of measured distance $d_{m}$ and the vertical distance Δh between the beacon and the mobile, as given by:

(22) $$d_{h}=\sqrt{d_{m}^{2}-\Delta h^{2}}.$$

### 6.2. Performance Evaluation

In this section, the performance of dead reckoning, Trilateration and Hybrid method are comprehensively evaluated. It is necessary to explain that the dead reckoning method requires the coordinate of the initial position. In each test, the person holding a smart phone continued to move along the determined path with a length of 28.8 m in the area covered by the BLE modules. In the experiment, the positioning performances of the three methods in a dense Bluetooth environment were tested.

#### 6.2.1. Dead Reckoning

**Figure 9:**
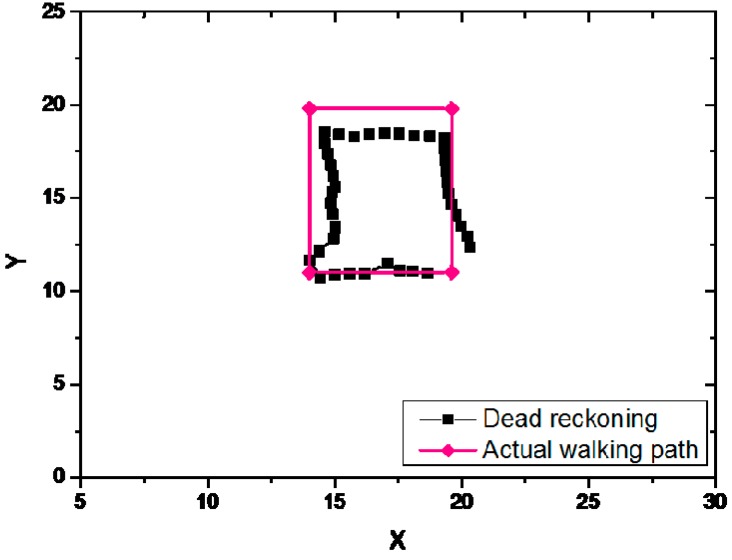
The estimated path and actual walked path of the dead reckoning.

In the dead reckoning experiment, the magnetic sensor and accelerometer of the smart phone were mainly used for positioning. The dead reckoning method should get its initial coordinate at the beginning of the position, because it cannot calculate the initial coordinate by itself. The estimated path and actual walked path of the dead reckoning is shown in Figure 9.

The estimated path of dead reckoning method was similar to the actual walked path. As the dead reckoning method requires initial coordinate to calculate the path, the cumulative error was increased with the increase of the estimated path, and a larger error appeared in the final location coordinate and the actual coordinate.

#### 6.2.2. Trilateration

**Figure 10:**
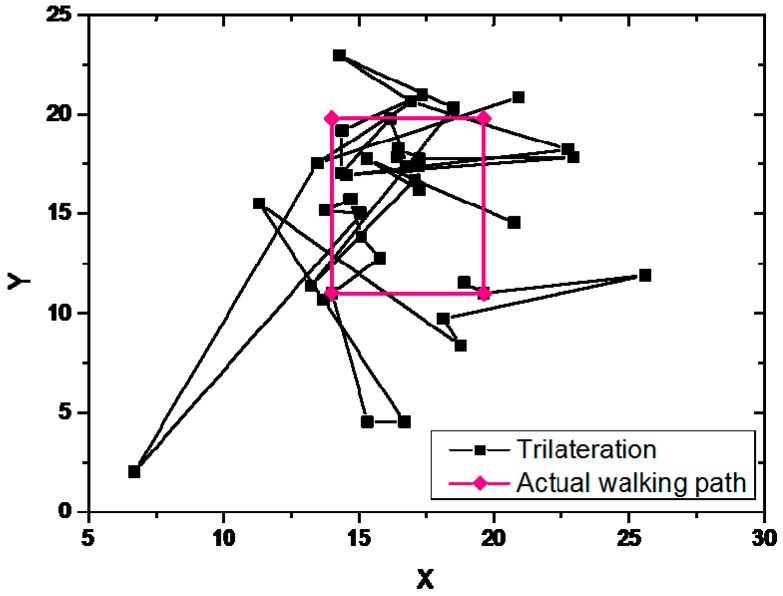
The estimated path and actual walked path of the trilateration.

The variation of the Bluetooth signal intensity becomes larger and the scanning interval of the BLE modules become longer under a dense Bluetooth environment. It had a great influence on the positioning accuracy of trilateration method. The estimated path and actual walked path of the trilateration is shown in Figure 10.

The estimated path of trilateration experiment was very different from the actual walked path The BLE positioning fluctuates resulting from fast fading or multipath effects in public indoor spaces. Due to the fast fading and multipath effects in the experiment environment, the trilateration method did not accurately position the actual location. The estimated path of the trilateration experiment was very different from the actual path. The experiment results showed that the trilateration method based on Bluetooth was difficult to independently complete indoor positioning with less error.

#### 6.2.3. Hybrid Method

**Figure 11:**
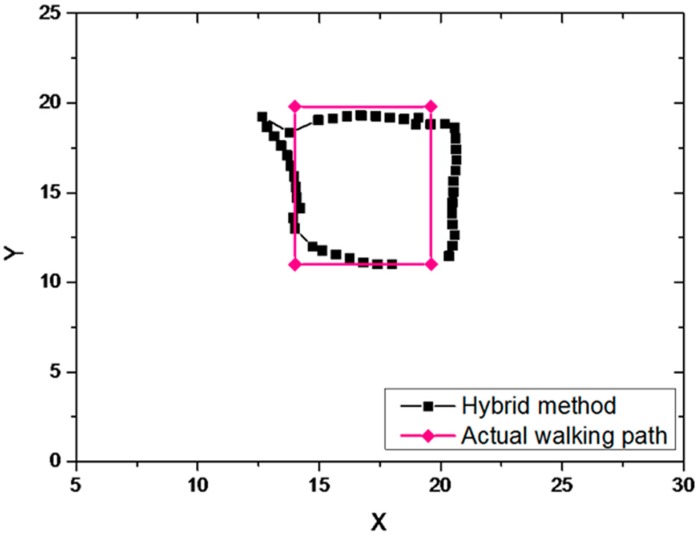
The estimated path and actual walked path of the hybrid method.

The hybrid method has the advantages of dead reckoning method and trilateration method. It can not only ensure that the estimated path has a small error with the actual walked path, but also can compensate the estimated path continuously to prevent a large cumulative error. The estimated path and actual walked path of the hybrid method is shown in Figure 11.

The estimated path of hybrid method was basically the same as the actual walked path. Under the premise of the estimation path calculated by dead reckoning method, the Kalman-based hybrid method effectively reduces the estimated path error and achieves better positioning results.

#### 6.2.4. Positioning Root-Mean-Square Error

The positioning root-mean-square-error (RMSE) of the above three positioning methods were calculated, as shown in Equation (23) [26]. The RMSE was used to evaluate the closeness of the measurement trajectory to a true trajectory over *N_step_* simulation steps. The results are shown in Table 5.

(23) $$RMSE\;=\;\sqrt{\frac{1}{N_{step}}\sum_{t=1}^{N_{step}}\left[{\left(x_{m}-x_{t}\right)}^{2}+{\left(y_{m}-y_{t}\right)}^{2}\right]}\;,$$

where ($x_{t},\;y_{t})$ is the true position coordinate at step *t*, ($x_{m},y_{m})$ is the measurement position, *N_step_* is the number of measurements of indoor positioning.

### 6.3. Results

According to the results of the experiment, the estimated path of dead reckoning method is similar to the actual walked path, and the dead reckoning method can realize indoor positioning without additional hardware. However, the dead reckoning method needs initial coordinates to locate, and its cumulative error increases with the length of the walking path.

The trilateration method requires BLE module as hardware support. Moreover, the individual trilateration method is easily influenced by the dense Bluetooth environment and has great influence on its positioning accuracy. Therefore, more data needs to be calculated to get more accurate positioning coordinates.

The hybrid method combines the advantages of dead reckoning method and trilateration method. The estimated path of hybrid method is basically the same as the actual walked path. The hybrid method not only has the advantages of the smooth estimated path, but also has the advantages of real-time correction of the location path, which minimizes the positioning error.

## 7. Conclusions

In this paper, the indoor positioning problems in the dense Bluetooth are mentioned. A high Bluetooth density of the environment would lead to a high RSSI variation and a long-time interval collection of BLE, which would lead to a low accuracy and a poor timeliness of positioning. To solve the problems, a hybrid method combined with trilateration algorithm and dead reckoning method was innovatively proposed based on a two-phase positioning strategy. A Kalman filter was used to integrate the trilateration algorithm and dead reckoning. A number of experiments were carried out to evaluate the accuracy and limitations of each method in a real implementation with a dense Bluetooth environment. The positioning root-mean-square error results proved that the hybrid method has improved the location accuracy and reliability in the dense Bluetooth environment.

## Figures and Tables

**Table 1:** RSSI value and the scanning time interval of the BLE.

	Number of Received	Average Interval/s	Average RSSI	Standard Deviation of RSSI	RSSI Max-Min
Dense environment	93	6.652	−61	7.7	37
Sparse environment	1109	0.542	−62	2.2	12

**Table 2:** Calculations of the loss path exponent *n*.

	BLE-1	BLE-2	BLE-3	Average
*n*	1.76	1.47	1.70	1.64

**Table 3:** Step measurements in different thresholds.

**Threshold**	1	1.5	2	2.5	3	3.5	4
**Step Count**	41	41	34	25	22	15	8

**Table 4:** Heading measurement values.

**Desired Heading**	0/360	45	90	135	180	225	270	315
**Average**	−1.25	−44.86	−90.82	−135.38	178.77	224.99	270.86	315.53
**Standard Deviation**	1.22	1.30	1.97	1.43	0.90	1.29	1.22	1.36

**Table 5:** The root-mean-square-error (RMSE) of the three methods.

Method	Trilateration	Dead Reckoning	Hybrid Method
RMSE	2.330 m	0.823 m	0.757 m
